# Probing the interactions of CdTe quantum dots with pseudorabies virus

**DOI:** 10.1038/srep16403

**Published:** 2015-11-10

**Authors:** Ting Du, Kaimei Cai, Heyou Han, Liurong Fang, Jiangong Liang, Shaobo Xiao

**Affiliations:** 1 State Key Laboratory of Agricultural Microbiology, Huazhong Agricultural University, Wuhan 430070, P.R. China; 2 College of Science, Huazhong Agricultural University, Wuhan 430070, P.R. China; 3 College of Veterinary Medicine, Huazhong Agricultural University, Wuhan 430070, P.R. China

## Abstract

Quantum dots (QDs) have become one of the most promising luminescent materials for tracking viral infection in living cells. However, several issues regarding how QDs interact with the virus remain unresolved. Herein, the effects of Glutathione (GSH) capped CdTe QDs on virus were investigated by using pseudorabies virus (PRV) as a model. One-step growth curve and fluorescence colocalization analyses indicate that CdTe QDs inhibit PRV multiplication in the early stage of virus replication cycle by suppressing the invasion, but have no significant effect on the PRV penetration. Fluorescence spectrum analysis indicates that the size of QDs is reduced gradually after the addition of PRV within 30 min. Release of Cd^2+^ was detected during the interaction of QDs and PRV, resulting in a decreased number of viruses which can infect cells. Further Raman spectra and Circular Dichroism (CD) spectroscopy analyses reveal that the structure of viral surface proteins is altered by CdTe QDs adsorbed on the virus surface, leading to the inhibition of virus replication. This study facilitates an in-depth understanding of the pathogenic mechanism of viruses and provides a basis for QDs-labeled virus research.

With remarkable photostability and brightness, quantum dots (QDs) have generated much excitement for their various potential applications in tracking viral infection in living cells^1^,^2^,^3^,^4^,^5^,^6^,^7^. Viral components labeled with QDs have been used as imaging probes to reveal the infection details and dynamic interactions between the viruses and cellular components^8^,^9^,^10^,^11^,^12^,^13^,^14^,^15^,^16^. For instance, a host-cell-assisted labeling strategy for enveloped viruses has been established based on the natural assembly process of viruses inside the host cells^17^. It was also reported that Streptavidin-conjugated QDs were used to label the nucleocapsid of recombinant baculovirus, achieving the real-time monitoring of the interaction of single virus with late endosomes and the subsequent nucleocapsid transporting into cell nucleus^18^.

Recently, the cytotoxicity and biosafety assessment of QDs have become a major research focus. A number of studies have reported the toxicity of QDs on cells and bacteria^19^,^20^,^21^. Li *et al.* found that CdTe QDs and CdTe/CdS/ZnS QDs can induce autophagy, which is a key factor leading to the decrease of metabolic activity of PC12 cells^22^. Liu *et al.* investigated the toxicity of CdTe QDs on hematopoiesis, and found that QDs with a smaller size showed greater hematopoiesis toxicity, and the generation of reactive oxygen species (ROS) may be one of the underlying causes^23^. Tsoi *et al.* proposed that QD cytotoxicity is induced by the production of ROS, which can damage cell membrane, cellular proteins, mitochondria, nucleus and DNA^24^. Peng *et al.* systematically analyzed the cytotoxicity, the cellular uptake efficiency and elimination of specific QDs, and found that the up-regulation of cell stress-related genes by QDs was closely related to the intracellular amounts of QDs, which is determined by the physicochemical properties of QDs^25^. Slaveykova *et al.* explored the effect of carboxyl-CdSe/ZnS core/shell QDs on metal decontamination capacity of *C. metallidurans*, and found that the presence of QDs induced the increase of Cu and Pb uptake by *C. metallidurans*^26^. Although QDs have been successfully studied in terms of cells and bacterial toxicity, the interaction between CdTe QDs and the virus remains largely unknown.

Pseudorabies virus (PRV), a herpesvirus that causes Aujeszky’s disease in swine, has been prevalent worldwide since its first discovery in 1920^27^. The broad host range as well as the high morbidity and mortality rates have caused tremendous economic losses in swine production all over the world^28^. PRV, together with HSV-1, HSV-2 and varicellazoster virus (VZV), belongs to the α-Herpesvirinae subfamily^29^. Due to its remarkable infectivity to synaptic connections of neurons, PRV is widely used not only as a model to understand α-herpesvirus biology but also as a neuroanatomical tracing tool^30^. In the present study, we investigated the possible effects of CdTe QDs on virus using PRV as a model and explored the related mechanism, aiming to gain insights into the pathogenic mechanism of viruses and provide a basis for QDs-labeled virus research.

## Results and Discussion

### Characterization of GSH-CdTe QDs and PRV

**Figure 1:**
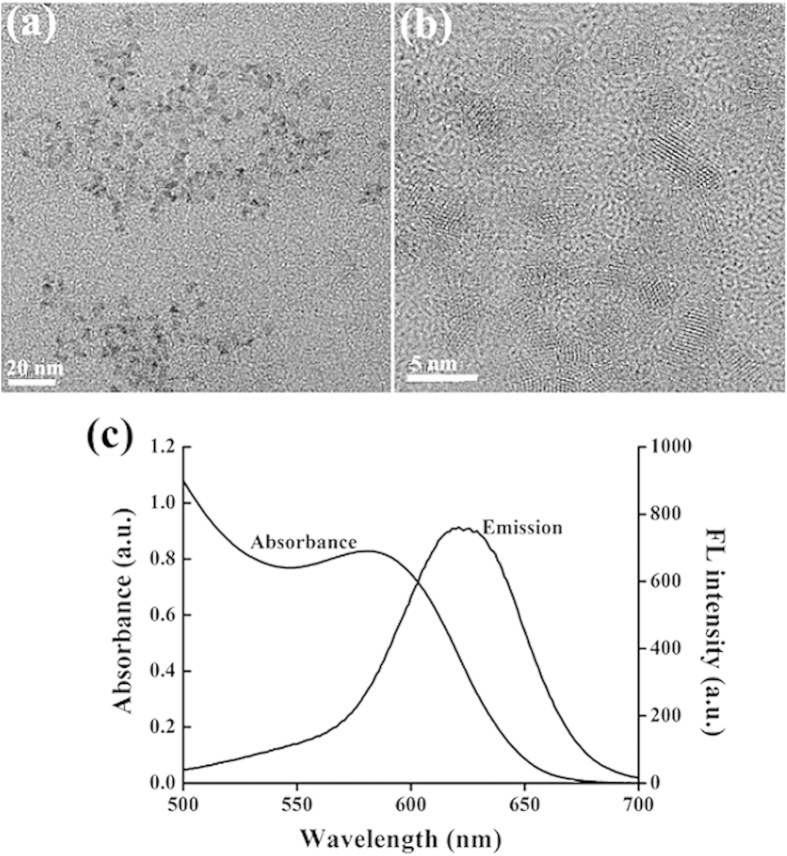
TEM (a) and HRTEM (b) of as-prepared GSH-capped CdTe QDs (624 nm). (**c**) UV-Vis absorption and fluorescence spectra of GSH-capped CdTe QDs.

As shown in Fig. 1a,b, transmission electron microscopy (TEM) and high resolution transmission electron microscopy (HRTEM) images confirmed that the as-prepared GSH-capped CdTe QDs were spherical particles with an average size of 3.2 ± 0.8 nm. The maximum fluorescence emission peak of CdTe QDs and the maximum absorption peak of CdTe QDs locate at 624 nm and 580 nm, respectively (Fig. 1c).

The hydrodynamic size distribution of PRV was analyzed by dynamic light scattering (DLS). As shown in Fig. S1, the average D_h_ of PRV was 190.1 nm (10^5^ PFU/mL), which is in agreement with the size of PRV previously reported^31^.

### Cytotoxicity of GSH-CdTe QDs on PK-15 cells

**Figure 2:**
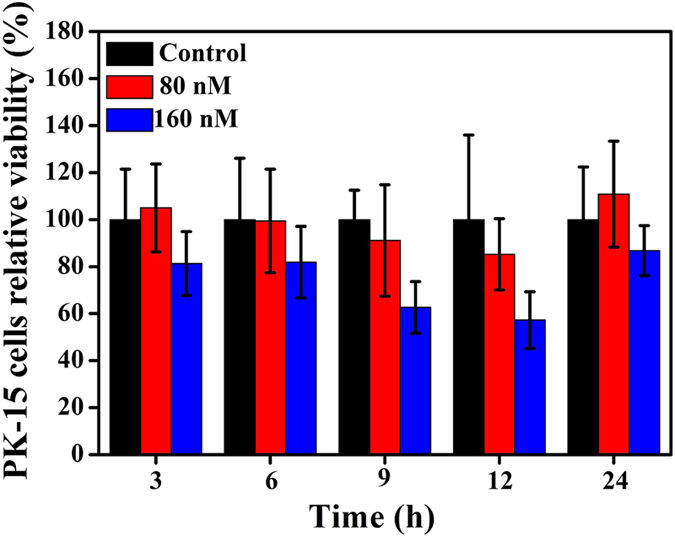
Cytotoxicity of different concentrations of CdTe QDs by MTT assay. PK-15 cells were incubated with CdTe QDs (624 nm) for 3, 6, 9, 12 and 24 h, respectively. Error bars represent the standard deviation from three repeated experiments.

To evaluate the potential cytotoxic effects of CdTe QDs on PK-15 cells, MTT assay was performed. Briefly, 80 nM and 160 nM CdTe QDs prepared with original materials were added to the cells cultured in 96-well plates and incubated for 3, 6, 9, 12 and 24 h, respectively. The results from Fig. 2 demonstrated that 160 nM CdTe QDs were highly toxic to PK-15 cells, whereas nearly no cytotoxicity was detected in the cells treated with 80 nM CdTe QDs. Therefore, 80 nM CdTe QDs were used in the subsequent experiments.

### Influence of GSH-CdTe QDs on PRV replication

**Figure 3:**
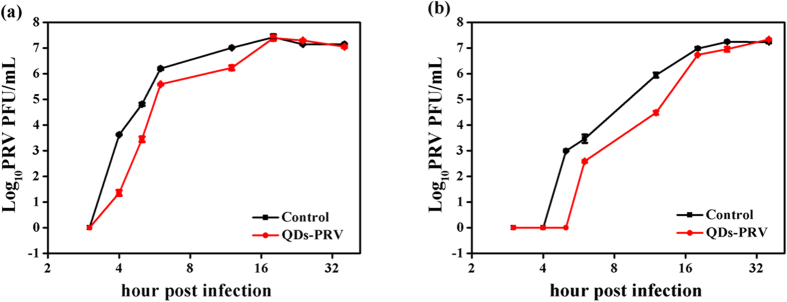
Growth curves of viruses in the absence and presence of 80 nM GSH-CdTe QDs (624 nm). PK-15 cells were infected with PRV at MOI = 2 for the indicated period of time. (**a**) The virus titer of intracellular and (**b**) The virus titer of supernatant. Error bars represent the standard deviation from three repeated experiments.

To clarify the influence of CdTe QDs on PRV replication, one-step single curve was examined in the presence of 80 nM CdTe QDs. The titers of intracellular and supernatant viruses pretreated with or without CdTe QDs were analyzed by plaque assays, respectively. As shown in Fig. 3a, the intracellular infectious virus, whether pretreated with CdTe QDs or not, was first detected at 4 hour post infection (hpi). However, when compared with the control, the PRV pretreated with CdTe QDs showed a remarkably decreased number of progeny infectious viruses in the early period of virus multiplication. At the end of virus multiplication (after 18 hpi), there was no apparent difference between the control and QDs-PRV. In Fig. 3b, the first infectious progeny virus in the control and QDs-PRV was detected at 5 and 6 hpi, respectively. When adsorbed on the surface of PRV, CdTe QDs may significantly delay the onset of progeny virus formation at the beginning of virus multiplication. In addition, CdTe QDs may also remarkably reduce the number of infectious progeny viruses in the early period of virus multiplication. However, the inhibitory effect of CdTe QDs on the progeny virus disappeared at the late stage of virus multiplication (after 18 hpi).

**Figure 4:**
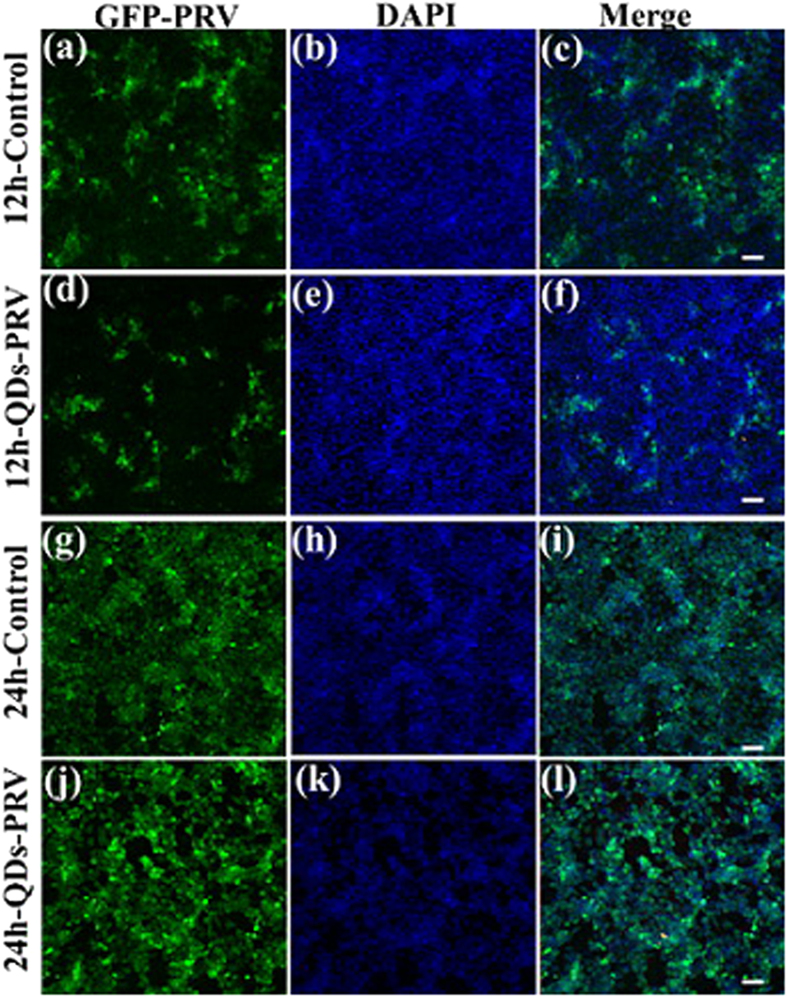
Fluorescence colocalization analysis of the control PRV and QDs-PRV on PK-15 cells. (**a–c**) Control: PK-15 cells infected with GFP-PRV for 12 hpi. (**d–f**) Fluorescent images of QD-GFP-PRV-infected PK-15 cells at 12 hpi. (**g–i**) Control: PK-15 cells infected with GFP-PRV for 24 hpi. (**j–l**) Fluorescent images of QD-GFP-PRV-infected PK-15 cells at 24 hpi. Scale bars: 50 μm.

The effect of CdTe QDs on PRV replication was further investigated by using a recombinant PRV expressing green fluorescent protein (GFP-PRV) at 12 and 24 hpi. A large number of green fluorescence signals were detectable with GFP-PRV at 12 hpi (Fig. 4a), but the number significantly decreased under the treatment of 80 nM CdTe QDs (Fig. 4d). These results indicated that a large amount of progeny viruses were suppressed at the early phase when the GFP-PRV was pretreated with CdTe QDs. However, no significant difference in GFP-PRV infection was observed between the treated group (Fig. 4g) and the control group (Fig. 4j) at 24 hpi. For further analysis, cellular nuclei were counterstained with 1.0 μg/mL of DAPI, and blue fluorescence signals were collected (Fig. 4b,e,h,k).

**Figure 5:**
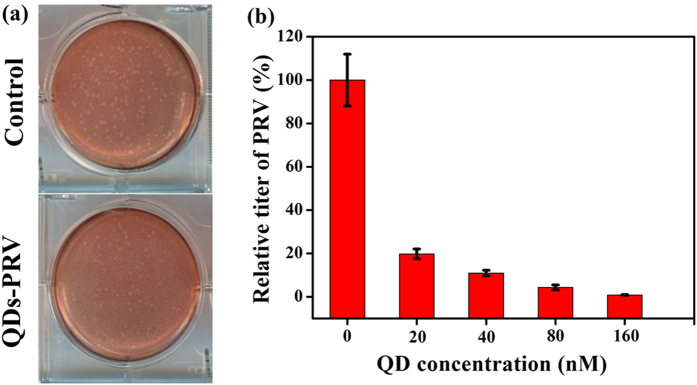
(**a**) Plaque assays of control and PRV pretreated with 80 nM CdTe QDs (624 nm). (**b**) The effect of CdTe QDs (624 nm) on relative titer of PRV. Error bars represent the standard deviation from three repeated experiments.

Entry process plays a key role in virus infection^32^. Fig. 5 shows the effect of CdTe QDs on the virus entry process, which demonstrates a significant dose-dependent inhibitory effect of CdTe QDs on PRV. It is well known that the entry of herpesvirus virions into cells is mediated by the viral glycoproteins^33^,^34^. First, the PRV virions are attached to the extracellular matrix by the interaction of gC with heparan sulphate proteoglycans, then the virion-cell interaction is stabilized by the binding of PRV gD to specific cellular receptors^35^,^36^, and finally, PRV gB, gH and gL mediate the viral envelope and the cellular plasma membrane fusion to release viral capsid and tegument into the cell cytoplasm^31^. The cell receptor binding sites were located on the surface of a virus, which is essential for virus attachment and entry into the host cells. It is possible that QDs on the surface of a virus can affect/inhibit the virus entry efficiency by combining its receptors and blocking its ligands^37^. Therefore, CdTe QDs may inhibit the virus entry by altering the protein structure on the virus surface.

**Figure 6:**
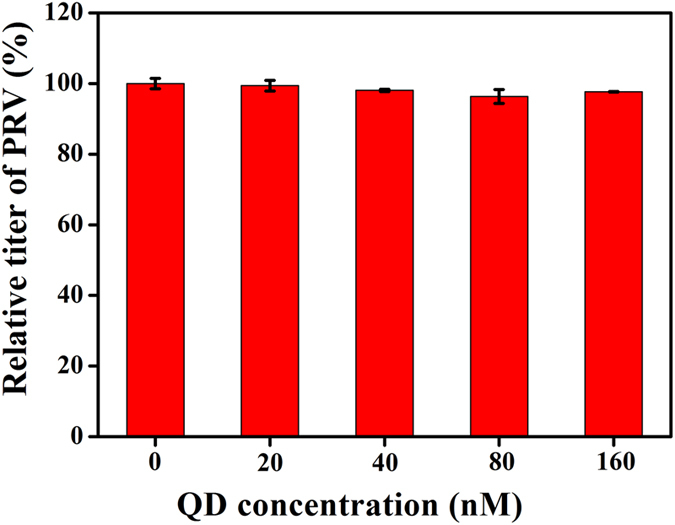
Effect of CdTe QDs on relative titer of PRV. CdTe QDs (624 nm) were added into the infected PK-15 cells. Error bars represent the standard deviation from three repeated experiments.

In order to investigate the effect of CdTe QDs on viral penetration, different concentrations of CdTe QDs were added to incubate with the PRV for 30 min after PRV adsorption on the cell surface for 2 h at 4 °C. As shown in Fig. 6, the titer of PRV did not show significant decrease with increasing concentrations of CdTe QDs. These results suggest that CdTe QDs can not inhibit the viral penetration, demonstrating that the effect of CdTe QDs on PRV proliferation is not the result of CdTe QD cytotoxicity.

### Effect of charge and size of QDs on the entry of PRV

**Figure 7:**
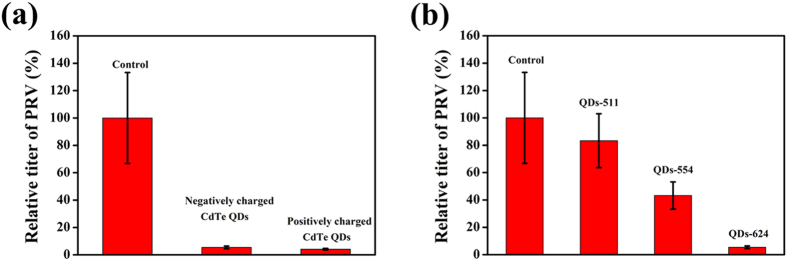
Effect of charge (a) and size (b) of CdTe QDs on relative titer of PRV during the virus entry process. Error bars represent the standard deviation from three repeated experiments.

In a previous study, we found that GSH-CdTe QDs had obvious inhibitory effect on the virus entry process, which is extremely significant for virus infection. To further characterize the factors that may influence the entry process, we tested the interaction of differently charged CdTe QDs with PRV. Zeta-potential measurements were performed using a Malvern zetasizer to determine the surface charge. GSH terminated with sulfhydryl groups, provides negatively charges on the surface of QDs, and the zeta-potential of GSH-capped CdTe QDs was −42.6 ± 6.32 mV. Positively charged QDs with zeta-potential value of 5.79 ± 0.66 mV was prepared by addition of ethylene imine polymer (PEI) to GSH-capped CdTe QDs according to the previously reported method^38^. Fig. 7a shows the effects of negatively and positively charged CdTe QDs during the virus entry process. It can be seen that positively charged CdTe QDs have more inhibitory effect than the negatively charged ones.

We also investigated the effect of QDs-511, QDs-554 and QDs-624 (Fig. S2) on the entry of PRV. As displayed in Fig. 7b, the relative titer of PRV decreased regularly as the size of GSH–CdTe QDs increased. This result is similar to that of a previous study on the effect of different sizes of CdTe QDs on human serum albumin^39^.

### Mechanism of interaction between QDs and viruses

**Figure 8:**
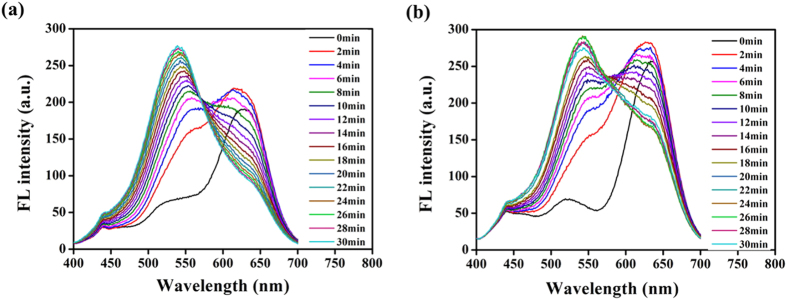
Fluorescence spectra of QDs-PRV and QDs-GFP-PRV. (**a**) GFP-PRV (10^5^ PFU/mL) was exposed to 160 nM GSH-CdTe QDs (624 nm) from 0 to 30 min. (**b**) PRV (10^5^ PFU/ml) was exposed to 160 nM GSH-CdTe QDs (624 nm) from 0 to 30 min.

The fluorescence spectra of CdTe QDs were recorded after the addition of PRV for different time intervals. As shown in Fig. 8, the maximum emission peaks of CdTe QDs are shifted to blue wavelength in the presence of PRV. It is well known that the sizes of CdTe QDs are related to their maximum emission peaks^40^. The blue shift of the maximum emission peaks indicates the decrease of QD sizes when PRV was added into the QD solution. When CdTe QDs are exposed to the PRV, they will be adsorbed on the surface of viral surface proteins. This process will change the surface properties and size of QDs, which is similar to the interaction of CdTe QDs with other proteins^39^. Lin *et al.* also observed a significant blue shift in the interaction between CdTe QDs and silk protein^41^.

The interaction between CdTe QDs and PRV also lead to the release of Cd^2+^, which was detected by Inductively Coupled Plasma Optical Emission Spectrometry (ICP-OES)^42^. The results indicated that 4.1 ± 0.3 μM Cd^2+^was released when PRV was exposed to 80 nM GSH-CdTe QDs (624 nm). The effect of Cd^2+^ on relative titer of PRV during the virus entry process was investigated. As shown in Fig. S3, a significant dose-dependent inhibitory effect was observed during the virus entry process. When the concentration of Cd^2+^ was 10 μM, the relative titre of PRV was 64.8%. However, the relative titre of PRV was just 4.3% after the treatment with 80 nM GSH-CdTe QDs (Fig. 5). These results indicate that CdTe QDs have stronger inhibitory effects than Cd^2+^ during the virus entry process, implying the inhibition of CdTe QDs on PRV can not be solely attributed to the toxic effect of Cd^2+^ release.

**Figure 9:**
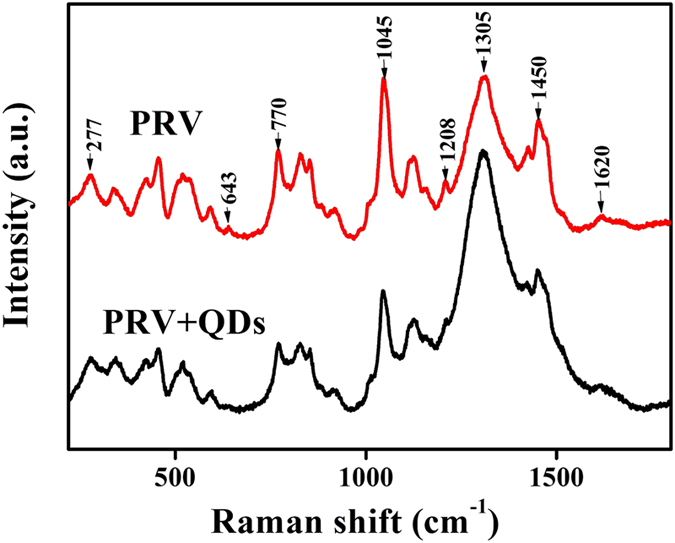
Raman spectra analysis of PRV and QDs-PRV. Black line is PRV (10^7^ PFU/mL) and red line represents PRV (10^7^ PFU/mL) pretreated with 80 nM CdTe QDs (624 nm). All Raman spectra were excited by 785 nm laser line and three times of accumulation.

Raman spectrum is a powerful tool to study the interaction of nanoparticles and biomolecules^43^. To further verify the interaction between CdTe QDs and PRV, an experiment focusing on the Raman shift of PRV and QDs-PRV was performed. PRV (10^7^ PFU/mL) was pretreated with 80 nM CdTe QDs (624 nm) for 1 h at 4 °C, and then analyzed by Raman spectra. Raman spectra of both PRV (control) and QDs-PRV are shown in Fig. 9 within the spectral region from 220 to 1800 cm^–1^. Obvious changes were observed in the shifts of 277 cm^–1^, 643 cm^–1^, 770 cm^–1^, 1045 cm^–1^, 1208 cm^–1^, 1305 cm^–1^, 1450 cm^–1^ and 1620 cm^–1^. The bands at 1208 cm^–1^ and 1305 cm^–1^ in the amide III region were due to the C-N stretching and N-H bending of the proteins^44^, and the spectra in the regions of 643 cm^–1^, 1450 cm^–1^ and 1620 cm^–1^ correspond to C-C twisting mode of tyrosine^45^, a CH_2_ bending vibration of the proteins^46^ and C=C stretching of proteins^47^, respectively. This result indicated that the protein structures were changed by the addition of CdTe QDs.

Circular dichroism (CD) spectroscopy is a powerful tool to study the conformational change of the secondary structure of protein molecules. To further verify the influence of CdTe QDs on the protein structure on virus surface, far-UV CD spectra of PRV in the absence and presence of CdTe QDs were analyzed. As shown in Fig. S4, the CD spectra of PRV have two negative bands at 208 and 222 nm, which are the characteristic spectra for α-helical structure of protein. The peaks at 208 and 222 nm are related to the π-π* and n-π* transition of peptide bonds in the α-helix, respectively^48^. The negative peaks of PRV were decreased after treatment with CdTe QDs, which also indicates that the structure of the viral surface protein was dramatically altered by CdTe QDs.

Based on the aforementioned results, it can be deduced that CdTe QDs may inhibit the proliferation of PRV through two possible models: after adsorption on the PRV surface, a) CdTe QDs change the structure of viral surface proteins and inhibit the PRV entry efficiency; and b) CdTe QDs results in the release of Cd^2+^, which leads to the decrease of the number of the viruses which can infect cells. Although the biological characteristics of the virus are affected by QDs, the cytotoxicity of QDs can be reduced by varying the particle size, shape, surface charge, hydrophobicity and the nature of the surface ligands to make QDs fit for labeling virus^49^,^50^.

## Conclusions

In this study, we demonstrate that GSH-capped CdTe QDs can inhibit virus multiplication by using PRV as a model. The inhibitory effect of CdTe QDs is not derived from the cytotoxicity, but more likely the result of the interaction between CdTe QDs and the virus. When adsorbed on the virus surface, CdTe QDs change the structure of viral surface proteins and subsequently lead to the release of Cd^2+^. This study facilitates the further understanding of the pathogenic mechanism of viruses and provides useful information for QDs-labeled virus research.

## Methods

### Preparation of GSH-CdTe QDs

Glutathione (GSH) capped CdTe QDs were prepared as previously reported with minor modifications^51^. Briefly, 0.3384 g CdCl_2_ and 0.5523 g GSH were dissolved in 150 mL deionized water, and the pH of solution was adjusted to 10.5 by adding 1.0 M NaOH solution. Then, the mixture was stirred under nitrogen for 20 min, followed by a rapid injection of 0.0630 g sodium citrate, 0.0655 g sodium tellurite and 0.6026 g sodium borohydride into the reaction flask one by one, and then the reaction mixture was heated to reflux for 6 h under nitrogen atmosphere. Next, the resulting CdTe QD solution was purified through precipitation from ethanol at the rate of 1:1, and the mixture was centrifuged at 8000 rpm for 10 min. Finally, the precipitate was resuspended in an equal volume of deionized water and stored at 4 °C for further use.

### Cells and viruses

PK-15 cells, a Porcine kidney cell line extremely permissive for PRV infection, were incubated in Dulbecco’s modified Eagle’s medium (DMEM) containing 10% fetal bovine serum (FBS) in a humid incubator with 37 °C/5% CO_2_. Pseudorabies virus (PRV) strain Ea (a wild virulent strain separated in China) was propagated in confluent monolayer of PK-15 cells in the presence of DMEM supplemented with 2% FBS at 37 °C for 2–3 days. When the cytopathic effect of PRV became apparent, the cell culture was submitted to a triple freezing/thawing cycle for a full release of the viruses, and the cell debris was filtered by centrifugation at 3000 rpm at 4 °C for 10 min^8^.

### Virus titer assay

The titer of PRV was determined by plaque assays as reported previously^52^. Briefly, PK-15 cells were incubated in 6-well plates in DMEM supplemented with 10% FBS for 24 h to reach 95% confluence. Subsequently, virus samples were 10-fold serially diluted (1.0 mL) with DMEM (2% FBS) and then injected into the confluent monolayer of cells for 1 h at 37 °C. After removing the inoculum and washing PK-15 cells three times with phosphate-buffered saline (pH 7.4), the cells were covered with 2XDMEM mixed with 1.8% low melting point agarose (Promega) at the rate of 1:1 supplemented with 3% FBS, followed by incubation in a 5% CO_2_ incubator for 3–4 days. Finally, PK-15 cells were stained with neutral red mixed with PBS at a 1:1 ratio for 2 h at 37 °C^53^. Virus titer was obtained by calculating the average plaque number from three independent experiments, and was presented by the plaque-forming unit (PFU/mL).

### MTT assay

10^4^ PK-15 cells per well were seeded in 96-well plates for 24 h to reach 100% confluence, and different concentrations of CdTe QDs were added to PK-15 cells at 37 °C. After 1 h incubation, the inoculum was removed and replaced with DMEM supplemented with 2% FBS (100 μL/well). After incubation for different periods of time (3 h, 6 h, 9 h, 12 h and 24 h), the supernatant was discarded, and 20 μL MTT reagent (3-[4,5-dimethylthiazol-2-yl]-2,5-diphenyl tetrazolium bromide, Sigma) was added to each well. After culturing for 4 h at 37 °C, MTT-reagent was removed and the formazan precipitate was dissolved with DMSO (150 μL/well). Absorbance at 570 nm was measured by using an Enzyme Linked Immunosorbent Assay (ELISA) microplate reader^54^. All measurements were done with a replicate number of eight wells per concentration. Viability was calculated by comparison with control cultures of normal PK-15 cells without exposure to CdTe QDs.

### One-step growth curve

80 nM CdTe QDs were mixed with PRV for 1 h at 4 °C, and then added to PK-15 cells at an multiplicity of infection (MOI) of 2 for 1 h. After infection for 1 h at 37 °C, the infected cells were treated for 60 s with pH 3.0 citrate buffer to stop the penetration of virions^55^. The infected cells were washed twice with PBS and then supplemented with medium (2% FBS) for further incubation. Medium supernatant and cell lysate were collected separately at 3, 4, 5, 6, 12, 18, 24 and 36 hpi, and stored at −80 °C. The supernatant and cell lysate were subjected to a triple freezing/thawing cycle to release viruses completely and the cell lysate was centrifuged at 3000 rpm at 4 °C for 10 min to remove cell debris. The infectivity of supernatants and cell lysate collected at different infection time periods was detected by plaque assays to establish a one-step growth curve^56^.

### Entry assay

PRV was mixed with different concentrations of CdTe QDs or Cd^2+^ at 4 °C for 1 h and then added into 6-well plates containing confluent PK-15 cell monolayers, followed by 1 h incubation at 37 °C/5% CO_2_ to allow PRV entry and infection. In order to inactivate the non-entryed virions, the medium was discarded and the cells were treated with pH 3.0 citrate buffer (60 s). The cell monolayers were gently washed three times with PBS to remove the citrate buffer^55^. Finally, cells were overlaid with 1.8% Bacto agar mixed with 2XDMEM (1:1) supplemented with 3% FBS^57^. The concentration of PRV was determined by virus titer assay.

### Fluorescence colocalization analyses

To verify whether CdTe QDs can inhibit PRV proliferation, PK-15 cells were infected with GFP-PRV and QDs-GFP-PRV at an MOI of 2. At 12 and 24 hpi, supernatants were removed, and cells were washed three times with ice-cold PBS and fixed with 4% paraformaldehyde for 15 min at room temperature. Next, the paraformaldehyde was discarded and ice-cold methanol (−20 °C) was added immediately to permeabilize the cells for 10 min at room temperature, followed by three times of rinsing with PBS, then staining with 1.0 mg/mL 4′,6′-diamidino-2-phenylindole (DAPI, Beyotime) for 15 min in dark room and then another three times of rinsing with PBS. All of the above steps were carried out in a shaker with the minimum speed^58^. Finally, the fluorescent images were visualized using a Zeiss LSM 510 Meta confocal laser-scanning microscope (Carl Zeiss Micro imaging GmbH, Germany).

### Penetration assay

The cell monolayers were incubated with PRV for 2 h at 4 °C, and after 2 h adsorption, the inoculum was removed and the cells were supplemented with DMEM (2% FBS) containing different concentrations of CdTe QDs, followed by 30 min incubation at 37 °C to initiate viral penetration^55^. Finally, the supernatant was discarded and the remaining steps were similar to those for entry assays.

## Additional Information

**How to cite this article**: Du, T. *et al.* Probing the interactions of CdTe quantum dots with pseudorabies virus. *Sci. Rep.* **5**, 16403; doi: 10.1038/srep16403 (2015).

## Supplementary Material

Supplementary Information
