# Supplementary material for: Probing the interactions of CdTe quantum dots with pseudorabies virus
Source: Sci Rep. 2015 Nov 10;5:16403. doi: 10.1038/srep16403 (PMC4639764; doi:10.1038/srep16403)
Supplement: Supplementary Information [file srep16403-s1.doc]

**Supplementary Information for**

**Probing the interactions of CdTe quantum dots with pseudorabies virus**

Ting Du^1,2^,*, Kaimei Cai^1,3^,*, Heyou Han^1,2^, Liurong Fang^1,3^, Jiangong Liang^1,2,#^ & Shaobo Xiao^1,3,#^

Transmission electron microscopy (TEM) images of QDs were taken by a JEM-2010FEF transmission electron microscope operating at an accelerating voltage of 200 kV (JEOL, Japan). Ultraviolet-Visible (UV-Vis) absorption spectra were recorded using Nicolet Evolution 300 UV-visble spectrometer (Thermo, USA). Fluorescence spectra were performed on a RF-5301PC (Shimadzu) fluorescence spectrometer. The hydrodynamic size distributions of PRV were analyzed by dynamic light scattering (DLS) technique using a Zetasizer Nano-ZS90 (Malvern Instruments Ltd., UK). Meanwhile, the zeta-potential of CdTe QDs was determined on a Zetasizer Nano-ZS90. Raman spectra of PRV were measured using an inVia Raman spectrometer (Renishaw, UK) equipped with a confocal microscope (Leica, German). Circular dichroism (CD) spectra were recorded by a J-1500 Spectropolarimeter (Jasco, Japan) under constant nitrogen flush.


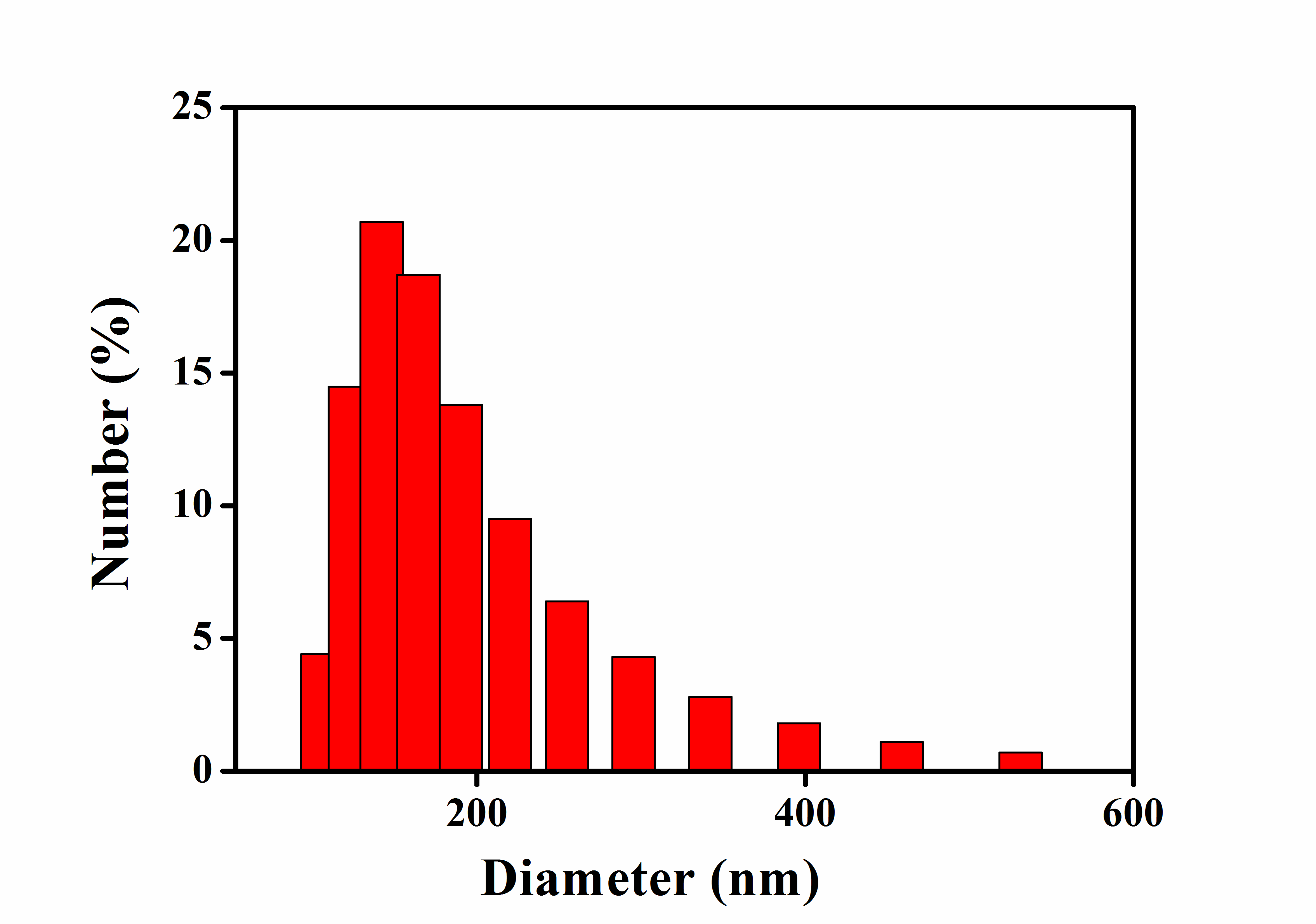


Figure S1. Hydrodynamic size distribution of PRV.

**
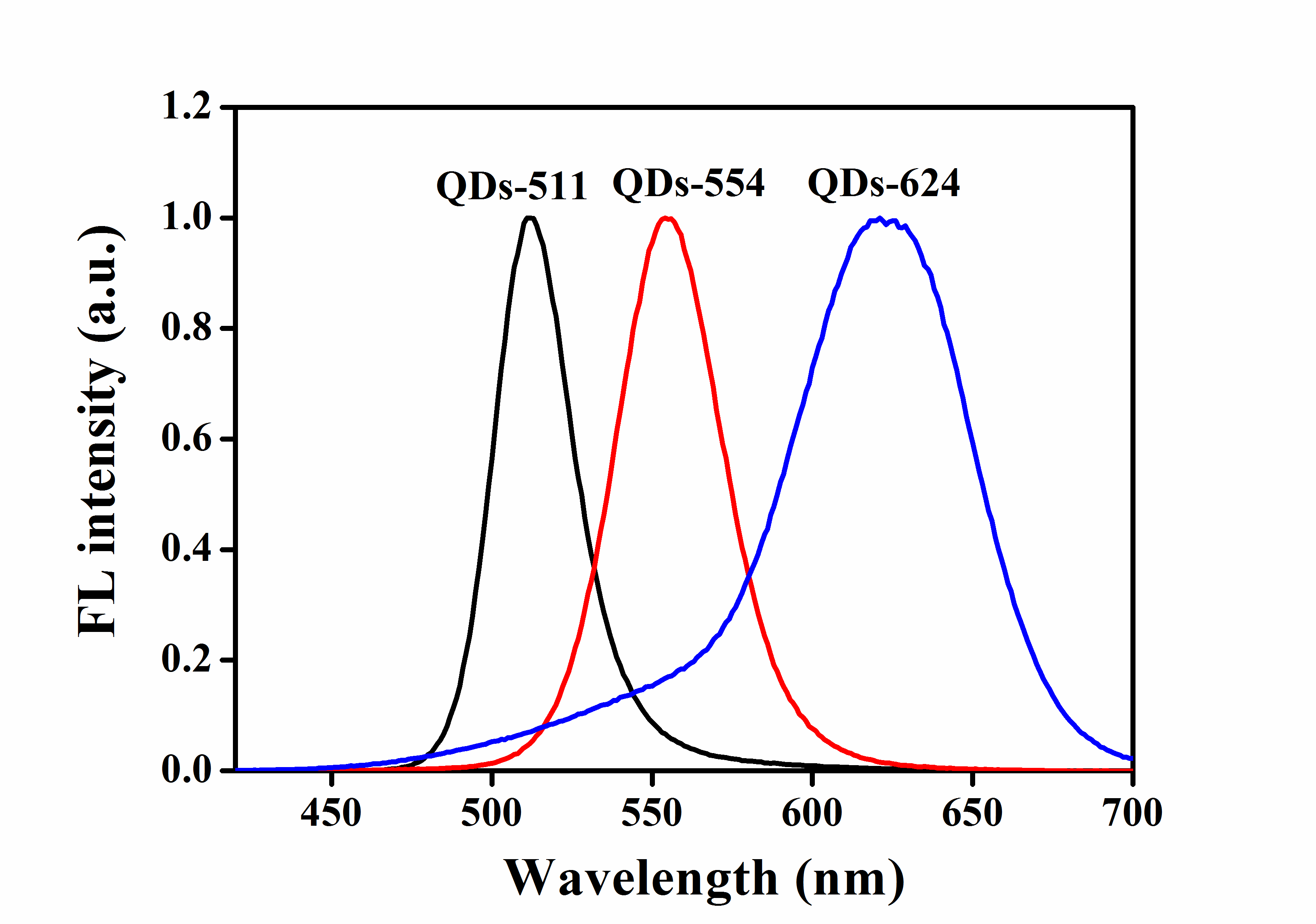
**

Figure S2. Fluorescence spectra of GSH-capped CdTe QDs. The fluorescence emission peaks are at 511, 554 and 624 nm, respectively. The sizes and concentrations of GSH-CdTe QDs were estimated from the ﬁrst absorption maximum of the UV-Vis absorption spectra by Peng’s empirical equations^1^. The sizes of the GSH–CdTe QDs were calculated to be 1.4, 2.8, and 3.5 nm, respectively.


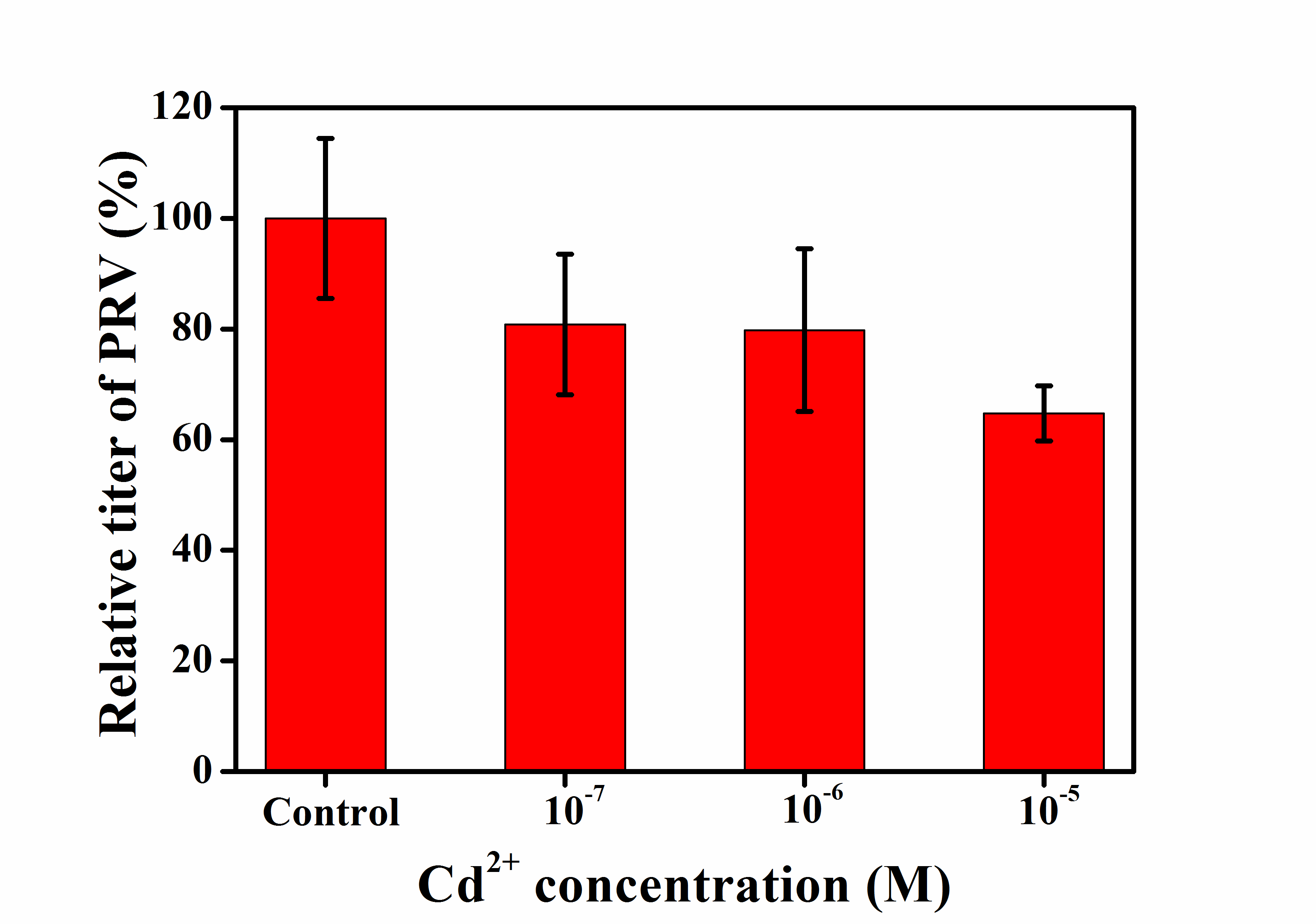


Figure S3. Effect of Cd^2+^ concentration on relative titer of PRV during the virus entry process. Error bars represent the standard deviation from three repeated experiments.


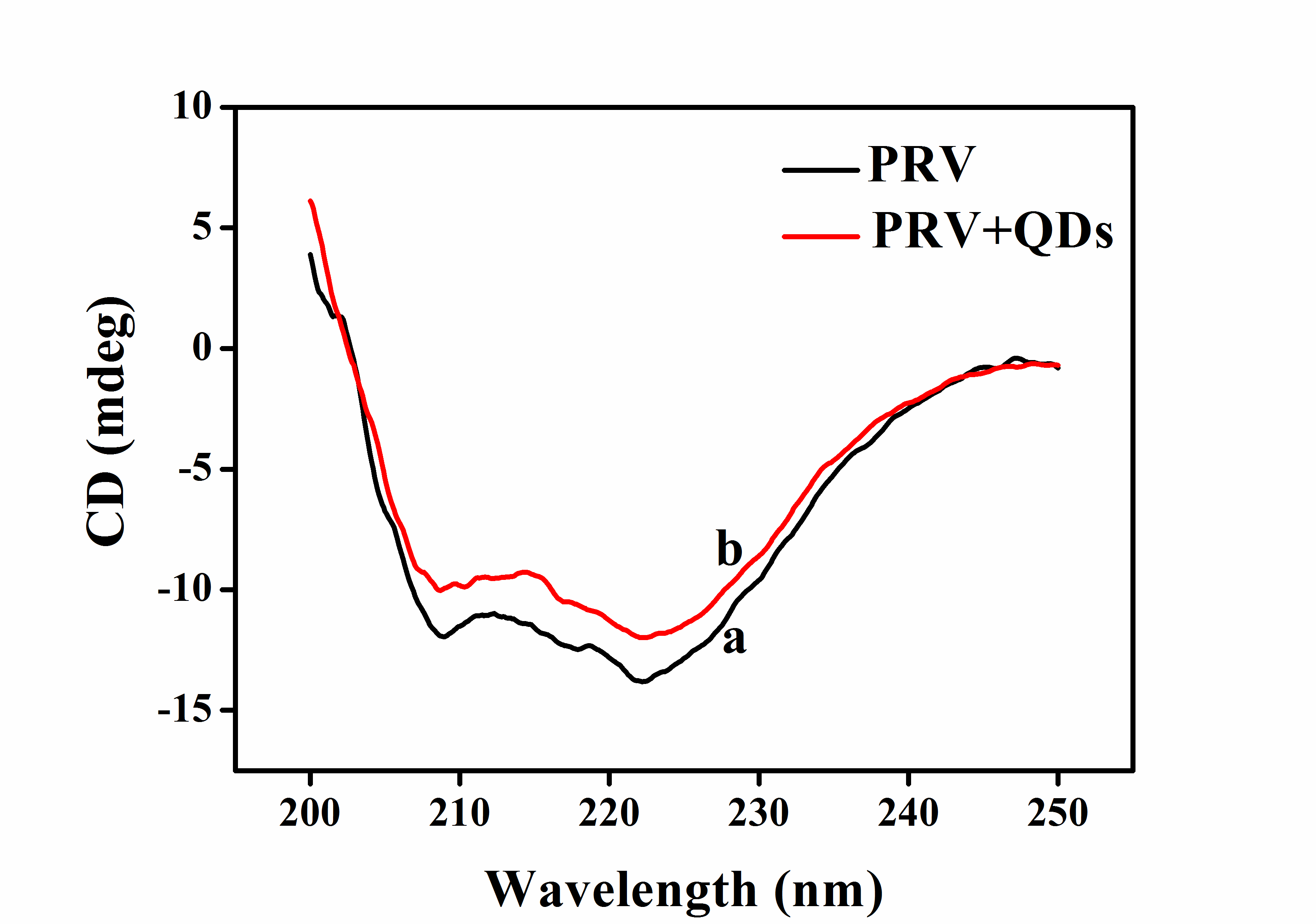


Figure S4. Far-UV CD spectra of PRV in the absence (a) and presence (b) of GSH-CdTe QDs (624 nm). The concentration of PRV was 2.0 × 10^5^ PFU/mL, and the concentration of GSH-CdTe QDs was 80 nM.

**Reference**

1. Yu, W. W., Wang, Y. A. & Peng, X. Formation and stability of size-, shape-, and structure-controlled CdTe nanocrystals: ligand effects on monomers and nanocrystals. *Chem. Mater.* **15**, 4300-4308 (2003).
